# Building consensus on identifying research mentoring gaps and finding ways of addressing the gap in a Kenyan college of health sciences

**DOI:** 10.4102/phcfm.v11i1.1886

**Published:** 2019-07-08

**Authors:** Masemiano P. Chege

**Affiliations:** 1 School of Medicine, Moi University, Eldoret, Kenya

**Keywords:** mentoring, research, Delphi technique, iterations, consensus, graduate students, academic members of staff

## Abstract

**Background:**

The concept of mentoring in clinical practice has traditionally focused on moving graduates from novice to more respectable positions within the clinical practice hierarchy. With the growing emphasis on evidence-based practice, the role of research in generating evidence for practice cannot be overemphasised. Mentoring in clinical operational research for both students and junior members of academic staff in health professionals’ training colleges is as important as mentoring for clinical skills.

**Aim:**

This study aimed at building consensus on possible ways of enhancing research mentoring for graduate students and members of academic staff in a college of health sciences.

**Setting:**

The study was conducted within Moi University College of Health Sciences (MUCHS) in Eldoret, Kenya.

**Methods:**

The study population was composed of academic staff members and registered graduate students by the end of 2015. All academic staff and graduate students were eligible to participate. The Delphi technique was used to not only collect individual opinions but also build consensus. During the first iteration, questions were sent for which open-ended responses were needed. Responses from the first round were grouped into patterns and themes that guided the writing of questions for the subsequent rounds.

**Results:**

The response rate was 78%. There was consensus in appreciating that mentoring was fundamental for career growth in clinical practice and research and needed for improving and developing formal structure for effective mentoring. It was crucial to establish training programmes for mentors and for accrediting them.

**Conclusion:**

Enhancing of current research mentoring in MUCHS was needed and expected by graduate students and academic staff.

## Background

Available data on the role of mentoring in medical practice and research reporting it as beneficial at each stage of training and associating it with greater research productivity, career retention and promotion. Most of these data are from high-income countries.^1,2,3,4,5,6,7,8,9^ Positive effects of well-structured mentoring programmes in graduate programmes have been documented.^10,11^

There is also evidence that early career mentoring for translational researchers is a process that aims at junior faculty members evolving from novice to expert researchers in a coordinated and monitored programme. This process has been most effective where experts and more experienced researchers apply tasks that are also common in other forms of human relationships. These include recognising compatibility between mentor and mentee, finding time for the needed activities, establishing patterns, agreeing on goals and ensuring that they are achieved.^4,7,12,13,14,15,16^

In most African and other low- and middle-income countries, resource constraints remain a big barrier for developing effective clinical and research mentoring infrastructure. There are, however, positive data that demonstrate that collaboration, between African medical schools and those that are less resource-constrained, is working towards infrastructural and financial support for the development of mentoring programmes in these African medical schools.^17,18,19^

According to the World Development Index (WDI) data on health systems by the World Bank in 2015, in Kenya, the doctor to population ratio was at 0.2 per 1000.^20^ In Kenya, nearly all postgraduate medical school training courses have a stringent compulsory research training programme that requires the candidate to write a thesis, which would be determined by graduate studies’ examiners, to obtain the postgraduate degree. The period of graduate training in Kenya offers a unique opportunity for research mentoring to nurture researchers in biomedical sciences.

The Moi University School of Medicine has an above average regional rating in research output within Eastern Africa.^21^ This is the result of a very fruitful partnership with the ‘Academic Model Providing Access to Health Care (AMPATH) Consortium’. The Academic Model Providing Access to Health Care is an educational medical partnership between North American academic health centres led by the Indiana School of Medicine and the Moi University School of Medicine, and has flourished in the time since it started in 1989.^22^ This collaborative effort has benefited health service delivery at the regional level in western Kenya where AMPATH and the Moi University collaborate with the county health departments to enhance chronic disease management within level three and four public health facilities. In the Moi University School of Medicine Teaching Hospital, the collaboration in clinical teaching, service and research has resulted in highly rated medical graduates. In research, the collaboration focuses significantly in translational research where research experts from partner universities in the west mentor Kenyans to grow from novices to expert researchers.

A pilot survey that interviewed graduate students, and junior and senior faculty members revealed that there was a significant satisfaction gap in research mentoring that needed to be addressed. It was agreed that the best approach to identifying and addressing the gap would be through consensus building and that the Delphi technique would be most appropriate for identifying and addressing the existing research mentoring gap in the Moi University College of Health Sciences (MUCHS).

### Broad objective

The aim of this study was to identify the research mentoring gap and find ways of addressing it for graduate students and faculty members in the MUCHS.

### Specific objectives

- To identify the research mentoring gap among graduate students and junior faculty members in MUCHS.
- To build consensus on ways to enhance research mentoring among graduate students and faculty members in MUCHS.
- To use evidence-based best practices through consensus to propose cost-effective methods to enhance research mentoring in MUCHS.

## Methods

### Design

We used the Delphi technique, which is a qualitative study design that facilitates a group communicator process that aims to achieve convergence of opinions on a specific real-world issue. This technique can use mixed qualitative and quantitative methods. During a feasibility survey, it was observed that some potential participants significantly controlled opinion during group discussions, when conducting in-depth interviews on faculty colleagues and it was realised that there was a possibility of compliance bias in the responses. The Delphi technique suited this study because there was no direct interaction among the respondents or even with the interviewers.

### Site

The study setting was MUCHS, Eldoret, Kenya.

### Study population

The study population comprised senior faculty members, junior faculty members and graduate students from MUCHS.

### Inclusion criteria

- Academic members of staff from different departments and schools on the December 2015 payroll in the MUCHS.
- Graduate students on the graduate students’ roll for the different departments and schools on the MUCHS in December 2015.

### Exclusion criteria

- Visiting academic staff from other universities at the time of the study.
- Academic members of staff and graduate students who declined to participate.

### Sampling procedures

The following three categories of participant included:

- Senior faculty members: senior lecturers, associate professors and professors
- Junior faculty members: lecturers and tutorial fellows
- Graduate students on the graduate students’ roll for the different departments and schools on the MUCHS in December 2015.

The three categories of graduate research work (senior faculty members, junior faculty members and graduate students) were conveniently selected to participate. This was guided by a survey carried out before the study when it was observed that all stakeholders (experts and learners) of research had differing opinions on ways to enhance research mentoring.

All senior members (senior lecturers, associate professors and professors) and junior academic staff from the different departments and schools were eligible to participate. A total of 460 participants (55 senior academic staff, 160 junior academic staff and 245 graduate students) were eligible to participate in the study.

### Data collection methods

Potential participants expressed support for the use of the email, with a Word document attachment, for receiving and sending responses.

For the first iteration, an open-ended question was mailed as a Word attachment to the eligible participants. The question read: ‘In your own opinion and observation, what does the Moi University College of Health Sciences (schools and departments) need to do to enhance research mentoring for faculty and graduate students?’

The responses of the first iteration would be grouped into themes within the three separate categories of participants and only those responses that had more than 50% concordance, among participants, would be used for the second iteration.

For the second and third iterations, consensus was set at an agreement of 80% and above. This was to minimise ambiguity.

For the third iteration, the same questions were sent out again to the same participants with highlights on themes that lacked consensus during the second iteration.

Regular reminders (by email, short mail messages by phone) were sent out every fortnight to participants who delayed responses during the first iteration, and those who did not respond after three reminders were declared as not consenting and lacking interest to participate in the first round.

The responses to the second and third iterations were prompt with minimal need for sending reminders.

### Data management

The responses to the first iteration question were analysed using pattern matching to develop themes that were used to prepare questions for the second and third iterations.

The responses to the questions of the second and third iteration questions were analysed as quantitative data and results presented in percentages.

Signed informed consent for all participants was a precondition to participation for the first iteration.

### Study implications

Building of consensus among research experts and teachers, junior faculty members and graduate students on cost-effective ways for research mentoring in MUCHS will influence policy and practice in MUCHS and other similar institutions.

### Study limitations

Our study aimed at interviewing all participants and so we did not have a scientifically representative sample. Without scientific sampling, it is possible that those who declined to participate may have a differing opinion, but with an above 75% response rate, the majority opinion can be considered as generally representative.

### Ethical considerations

Ethical approval was obtained from the Moi University Institutional Research Ethics Committee (IREC) and permission to conduct the study was obtained from the Principal of the Moi University College of Health Sciences (Formal approval number: FAN: IREC 1488).

## Results

The first iteration question was sent out to 55 senior faculty members, 160 junior faculty members and 245 postgraduate students.

Those who consented to participate and send responses back were 356 (45, 123 and 188) for senior, junior faculty members and graduate students, respectively. This was a response rate of 82%, 77% and 77% for the three categories of participants.

The responses from the three categories of participants to the open-ended questions of the first iteration are summarised in Tables 1–3.

**TABLE 1:** Summary of first iteration responses by senior faculty members.

Senior faculty expectation of graduate students	Expectations of senior faculty on junior faculty	Both junior and senior faculty
All should understand that mentoring is fundamental for career growth, promotion and retention in clinical practice and research Students should write project proposals in line with research interests of the faculty to enhance effective supervision and increase chances of publication	All should understand that mentoring is fundamental for career growth, promotion and retention in clinical practice and research Actively apply for research grants and seek guidance from the seniors	All should understand that mentoring is fundamental for career growth, promotion and retention in clinical practice and research The mentor and mentee relationship should be close and mutually acceptable at every step
A forum in the schools should be created where teachers present research ‘grey’ areas with high research impact to graduate students Students should also be encouraged to present on their areas of research interest during the same forum	Junior faculty should be encouraged to present their research interests within their departments for guidance Faculty members who are unable to develop their own independent research interests should be encouraged to attend refresher courses (even with graduate students) or be guided by seniors within the department	The need for a forum within the college where senior faculty present their work and all attend (senior and junior faculty and graduate students)
Introduction of formal mentorship unlike the current emphasis on research supervision Students to choose own mentors and supervisors Students to be mentored by working within projects of the mentors as opposed to students doing their own project or thesis	Seniors to mentor juniors Frequent workshops on mentorship Promotion points on effective mentoring	Allow change of supervisor anytime the student or supervisor is uncomfortable in continuing the relationship. No negative consequences Allow interests to guide mentor and mentee with the choice of who to work with
Relevant journals to be made available in departments Improvement of infrastructure and study facilities Logistic support to attend and participate in scientific conferences and other open forums	More PhD and clinical fellowship programmes should be developed in the college as venues for mentoring Logistic support to attend and participate in scientific conferences and other open forums within and outside Kenya	Encourage more research exchange programmes Recognition and merit awards on good mentor–mentee relationships Avail research grants Linkages and collaboration in teaching, research and learning
Proactive assigning of mentors and mentees Faculty training on mentorship Protected time for mentoring interaction Graduate students to be involved in research projects by senior faculty and use them for their dissertation. PI as mentors with relationships continue after graduation	Training on mentorship Assigning mentors to junior faculty upon employment Set aside small grants for the faculty members to help in mentoring Enhance collaboration for mentoring	Encourage a research culture with schools and departments Training on mentoring Continuous feedback on mentoring progress Building relationships that enable a conducive mentoring environment
Need for a conducive environment for research Research infrastructural enhancement (equipment and tools) Need to ‘infuse’ graduate students with research appreciation and its needs	Need for creation of a research environment that nurtures research culture Need for infrastructural enhancement (equipment and other resources) Need for enhanced and structured research funding Create collaborative linkages between universities and research institutions Encourage deserving researches with awards and other recognition	As above
Moi University to fund MMed, MSc and PhD research as part of tuition Regular meetings between teachers and learners (at least once a month) Relationship between student and teacher to be guided by focus on student benefit and on ‘equal’ terms or levels Need to address the various ‘curiosities and energies’ of the mentors [*sic*] Collaboration and networking among departments and their students	Need for continuous mentoring to build skills in research Departmental and school and college workshops to brainstorm on research ideas and areas of interest Staff motivation by the college needs to be enhanced Formal training on mentoring to be introduced Need to identify new or novel questions within departments that may raise departments to global recognition	As above
Research skills foundation should be laid down well for beginners to equip them with the needed research skills and minimise the time taken to develop a research proposal The institution should have proper time guidelines for research, for example, for promotion from first to second year, all research proposals should be presented at the same time such as within a calendar week with wide audience (students, faculty and other research experts) Enhance mentorship development for the above to succeed	Members who have few research publications and research experience should be partnered with those with more	As above
Having a good or large pool of academic staff who participate actively in research Organised research seminars held at least quarterly within MUCHS Collaborative research involving researchers from partners across the oceans Students to choose their supervisors and mentors. Departments to protect popular mentors from overload Easy access to journal articles	Departments to hire persons who do little other than research and use them as mentors for junior faculty Regular workshops to be started where faculty present their research activities. The workshops to include faculty and students Collaborative research among institutions Capacity building in research and research mentoring	Protected time for mentors and mentees to interact needed Formal research teams within departments and schools and colleges Graduate students and their mentors to be allocated time during which they present their work to other staff and faculty The departments and schools and colleges to reward and appreciate students and faculty members that do well
Journal clubs participation where journal articles are reviewed and critiqued Monthly meetings with faculty to discuss progress on research and identify and resolve any difficulties Attend workshops on research and make presentations, scientific conferences	Journal clubs participation where journal articles are reviewed and critiqued Schools should develop guidelines on scientific research proposal development Departments should allocate specific time for research presentations Invitation of resource persons to talk on research within schools and departments Include students in research projects	Mutual respect for mentor and mentee Observe timelines for submission of work and stick to them Each party to diligently do their part
Departmental libraries with basic research books, statistics books and other necessary resources Graduate students’ study rooms within departments Reliable internet connection	A common research office in the school. The office should be equipped with a computer, scanner and printer for ease of interaction between faculty and graduate students	A common research department and library within the school and college A common research office in the school. The office should be equipped with a computer, scanner and printer for ease of interaction between faculty and graduate students
Increase the number of graduate faculty to make them more available Have a fixed period and time on the timetable for research Increase the research courses and have these courses offered more than once (September–December) on admission. Could be taught several times during the MSc/MMed/PhD programme Develop a research fund for graduate students	The school to organise regular refresher courses on the supervision of research Moi University to provide funding to facilitate research Members of departments to jointly write grants for research funding. This would increase chances of success Include graduate students in faculty research activities	Regular joint research seminars to share new ideas and progress on ongoing research or studies

MUCHS, Moi University College of Health Sciences; PI, principal investigator.

**TABLE 2:** Summary of first iteration responses by junior faculty members.

Junior faculty expectations of graduate students	Junior faculty expectations of senior faculty	Both junior and senior faculty
The learners should be involved in choosing their research mentors unlike the current state where they supervisors are assigned to them Assigned projected time for mentoring interaction with students Students should actively seek and express interest in getting involved in research projects that individual faculty members are involved in All should understand that mentoring is fundamental for career growth, promotion and retention in clinical practice and research	All should understand that mentoring is fundamental for career growth, promotion and retention in clinical practice and research The need for formal and structured training on mentoring Assigning mentors to junior faculty members as soon as recruited into schools and departments Set aside small grants for faculty members to help with mentoring skills Enhance collaborations to purposefully target mentoring of faculty members	All should understand that mentoring is fundamental for career growth, promotion and retention in clinical practice and research Explicitly adopt a research culture in departments and schools Training on mentor and mentee relationship Continuous and structured two-way feedback on mentoring progress Building relationships that enable a conducive mentoring environment
Research should be a guided process where the supervisor and the graduate student have a friendly learning environment that is non-threatening with the supervisor offering professional guidance while allowing the registrar to bring out his or her thoughts. The mentor should offer support during challenging times, for example, offer feasible suggestions on addressing difficult reviewer comments. The mentor should make available any possible research grants to the mentee or advise when the research topic can attract research funds. The supervisor should be accessible during the research period and get constant briefs on the research process At the completion of the research process with data and results analysed, the supervisor and mentor should maintain professional contact and be in constant communication on research matters and any other relevant professional assistance more so on the processes involved in publishing results of the study Departments should at least have a research agenda where graduate students can engage and participate in research with their mentors. Departmental research is one way the graduate students can observe their mentors actively engaged in research and learn from their mentors how they manage challenges encountered during the research process	The research capacity of faculty members should be enhanced through workshops like ‘grant writing’ workshops. It would be a good thing to have ongoing research either departmental or collaborative research with other universities where new or junior faculty members with very little research experience can be incorporated and mentored by their senior colleagues. This is important because the research world is not a walk in the park; indeed, the research road is riddled with numerous challenges. Attracting research grants as a novice is not an easy task either and you have to belong to a ‘members club’. Overcoming some of these challenges requires a helping hand. These helping hands are senior faculty members with research experience. Research mentorship programmes within the faculty may go a long way in fostering this	Build research capacity for faculty and graduate students Actively run research mentorship programmes within departments Avail research grants and funds at college level even if possible at the departmental level incorporating research funds in the departmental budgets Support publishing frameworks, for example, reviewers network and publishing journal networks

**TABLE 3:** Summary of first iteration responses by graduate students.

Graduate students expectations of fellow graduate students	Graduate students expectations of faculty	Graduate students expectations of faculty and students
Have a refined research question that can easily be answered by the set objectives Allocate adequate time for research-related activities Keep or follow research-related timelines Consult with supervisors more frequently	Offer guidance on research question(s) Offer more guidance on the research proposal with more attention to the research methodology Involve graduate students in faculty research as a means of mentoring	Team work
An earlier communication on the need to conduct research as a student Make students aware of the need to self-sponsor themselves during research Steps to undergo before finally conducting the research	Provide prior information on the need to mentor students through research With earlier communicated areas of interest, the faculty members should be allocated students under their areas of specialisation or interest To have a clearly stipulated time schedule of when to meet deadlines as pertains to student efforts To pair up faculty members with the same interest and ideas to create feasibility on the student side in terms of a common mind and expectation To be allowed to declare their availability for the students during the entire period of mentorship	To allow students and faculty members to meet and agree on the required terms of working Disclose to both on source of funding for the research work Clearly stipulate the role and expectations from each

Only responses that had above 50% consensus were included in developing the themes that guided the second and third iterations. This was done to minimise inclusion of equivocal responses.

For Tables 1–3, the following apply:

- The different rows present collated themes of responses collected
- Different columns represent collated themes of responses about what may be needed to enhance research mentoring for the specific group
- Tables 1–3 represent collated themes of responses on what each group proposed should happen to enhance mentoring for each of the stakeholders.

These responses were then merged along common themes and used to develop closed questions for the second and third iterations. The summary of these responses is summarised in Tables 4 and 5.

**TABLE 4:** Summary of responses in the second iteration.

Ways to enhance research mentoring	What Moi University College of Health Sciences should do or not do?				
	MUCHS (should do)		MUCHS (should not do)	
	Frequency	%	Frequency	%
Mentoring is fundamental for career growth, promotion and retention in clinical practice and research and must be embraced by all	356	100.0	0	-
Graduate students to be involved in faculty grants and other research projects. This will be an opportunity for a long-term mentor and mentee relationship	356	100.0	0	-
The need for a forum (at school and college level) where research ideas or topics of interest and uncovered areas are shared across the departments and schools	356	100.0	0	-
Formal training in mentoring for faculty and graduate students (graduate students play a part in choosing their mentors)	320	90.0	36	10.0
Points to be awarded for effective mentoring and points to be awarded for promotion of faculty†	256	72.0	100	28.0
Formal introduction and declaration of research mentorship as opposed to the current research supervisor arrangement†	219	61.5	137	38.5
The need to develop infrastructure that favours research (more space for student to interact among themselves and faculty members) within departments	356	100.0	0	-
Facilitate students and faculty members to attend and present in scientific conferences (local and international)	356	100.0	0	-
Promote interest in reading research materials by providing relevant journals within departments	292	82.0	64	18.0
Departments to employ persons who do mostly research (and little else) and use them as research mentors†	128	36.0	228	64.0
Recruit more graduate faculty members to make them more available to mentor and teach research and avail blocked time for research training and implementation for graduate students and faculty	356	100.0	0	-
Enhance collaborations with other universities that promote mentoring teaching or learning and research	356	100.0	0	-
The need for training on research supervision	356	100.0	0	-

MUCHS, Moi University College of Health Sciences.

† , Consensus threshold of 80% not achieved.

**TABLE 5:** A summary of responses in the third iteration.

Ways to enhance research mentoring	What Moi University College of Health Sciences should do or not do				
	MUCHS (should do)		MUCHS (Should not do)	
	Frequency	%	Frequency	%
1. Mentoring is fundamental for career growth, promotion and retention in clinical practice and research and must be embraced by all	356	100.0	0	-
2. The need for formal structures for effective mentoring Initiate formal mentor(s) and mentee(s) programmes for graduate students and junior faculty members Establish modes of recognising and rewarding effective mentoring Establish formal local and international mentee and mentor exchange programmes	356	100.0	0	-
3. The need for formal accredited training for mentors with categorisation based on an achievement performance record	356	100.0	0	-

MUCHS, Moi University College of Health Sciences.

### The three items were presented as a summary of the responses in the second round for the third round (iteration)

By the end of the third iteration, we had realised the consensus from the participants.

## Discussion

Consensus by participants drawn from the graduate students, junior and senior faculty members of the MUCHS in Western Kenya was remarkable. By the end of the third iteration, the potential benefactors (senior faculty members) and beneficiaries (graduate students and junior faculty members) had unanimously agreed on ways needed to enhance research mentoring in this college that is located in a sub-Saharan African country.

By the third iteration the participants involved concurred that mentoring in research was requisite for career growth, promotion and retention in clinical practice and research. This crucial observation has been documented in studies that looked into mentoring in research and clinical practice. Most of the data are from developed countires.^1,3,4,5,6,7,8^

The important role played by mentoring in faculty development was supported by all junior faculty members, senior faculty members and graduate students and proposed as one that needed enhancing and structuring. There are available data that also support mentoring as crucial in faculty development.^6,23,24,25,26^ Decastro proposes a focus on developing a network of mentors geared towards individual needs of mentees instead of a theoretically assumed hierarchy of needs. This was also ranked high in our study.^23^

The participants in our study acknowledged that the current general assumption is that mentoring was going on in the MUCHS. There, however, was consensus on the need for structured mentoring that suited both the mentors and the mentees. Sambuco presented it more explicitly as ‘[an] Academic medical faculty often lacks the skills and knowledge necessary for successful negotiation, especially early in their careers, for effective mentoring to take place’.^27^ There are also other available data that also emphasised the need for structured mentoring programmes.^10,11,28,29^

Mentoring like any other academic engagement requires formal training and preparation of the mentors. The assumption that the successful careers of men and women make them effective mentors for those who look up to them for guidance has not worked to the satisfaction of the mentor and the mentee. One of the causes attributed to the poor results is the lack of well-trained and well-supported mentors. Abedin et al. proposed the need for validation and development of competencies for mentors.^16,30,31,32^

Our study participants proposed mentoring programmes that involved mentoring retreats, a mentoring consultation online (that included Kenyan and foreign experts). The same was presented by Fieldman et al. among other available documents on the subject.^16^

## Conclusion

Our study findings on methods that would enhance research mentoring concurred with findings documented in developed countries. Although MUCHS encourages mentoring as part of training and faculty development, there was a need for formal structuring of mentoring programmes and finding ways to appreciate successful mentor and mentee programmes.
